# Exposing an “Intangible” Cognitive Skill Among Collegiate Football Players: II. Enhanced Response Impulse Control

**DOI:** 10.3389/fpsyg.2018.01496

**Published:** 2018-08-22

**Authors:** Theodore R. Bashore, Brandon Ally, Nelleke C. van Wouwe, Joseph S. Neimat, Wery P. M. van den Wildenberg, Scott A. Wylie

**Affiliations:** ^1^Department of Neurosurgery, University of Louisville, Louisville, KY, United States; ^2^Department of Psychology, University of Northern Colorado, Greeley, CO, United States; ^3^Department of Psychology, University of Amsterdam, Amsterdam, Netherlands

**Keywords:** impulse control, Simon task, football, athletes, inhibition, attention

## Abstract

American football is played in a dynamic environment that places considerable demands on a player’s ability to make fast, precise reactions while controlling premature, impulsive reactions to spatial misinformation. We investigated the hypothesis that collegiate football players are more proficient than their non-athlete counterparts at controlling impulsive motor actions. National Collegiate Athletic Association (NCAA) Division I football players (*n* = 280) and non-athlete controls (*n* = 32) completed a variant of the Simon conflict task, which quantifies choice reaction speed and the proficiency of controlling spatially driven response impulses. Overall, the choice reaction times (RTs) and accuracy rates of football players and controls were equivalent. Similarly, football players and controls were equally susceptible to producing incorrect impulsive motor responses. However, the slowing of RT attributed to the activation and successful inhibition of these impulses (i.e., the *Simon effect*) was reduced significantly among football players compared to controls. Moreover, differences in *impulse control* varied by position among the players, with the reduction being greater for offensive than for defensive players. Among offensive players, running backs, wide receivers, and offensive linemen had greater *impulse control* than did controls, whereas among defensive players only linebackers had greater control. Notably, the *Simon effect* was reduced by 60% in running backs compared to controls. These results contribute to emerging evidence that elite football players possess more proficient executive control over their motor systems than their age counterparts and suggest that the speed of controlling impulsive motor reactions may represent an enhanced cognitive “intangible” among football players.

## Introduction

The physical skills of football players at the collegiate and professional levels are demonstrably more advanced than the physical skills of comparably aged young men who are not competitive athletes and have been recognized as such for decades. These differences in physical skills are certainly reflected in the extraordinarily small proportion of high school football players who go on to play at the collegiate level. Only 2.7% of the more than 1 million high school football players will compete at the top collegiate level [Division I (DI)] and only 1.6% of all collegiate football players (Divisions I, II, and III) will play at the highest professional level, the National Football League (NFL)^1^. Very few studies have been done, however, to determine whether football players also possess enhanced cognitive skills compared to their male counterparts who are not competitive athletes. Despite the dearth of contemporary research in this problem area, comparative studies of collegiate football players with other collegiate athletes (e.g., baseball, basketball) and non-athletes from the general student body can be traced to the 1920s (e.g., Bean, 1927; Burley, 1944; Keller, 1942). In addition, there have been a handful of studies in which the speed of an array of reactive actions considered essential to success in football have been studied with no comparisons to either an athlete or non-athlete control group. For example, charging speed (reaction speed out of a three-point stance in response to an auditory signal) and different influences on it (e.g., signal cadence rhythm and speed) have been assessed in college football players either without determining position differences in speed (Elbel et al., 1952) or by determining these differences (Miles, 1931; Miles and Graves, 1931; Thompson et al., 1958). Similarly, the relationship between college football players start times out of a three-point stance, their times in the 40-yard dash, and their body weight composition have been explored (Crews and Meadors, 1978). Recently, differences in visuomotor reaction time (RT) between high NFL draft picks and current roster players have been studied by Solomon et al. (2013) as part of a comprehensive neuropsychological assessment.

The majority of cognitive research completed to date on football players has been designed to identify neurocognitive sequelae in players who, for example, (i) have experienced single or multiple concussions as college (Ledwidge and Molfese, 2016; Mrazik et al., 2016) or professional (Goswami et al., 2016; Mrazik et al., 2016) players; (ii) have been exposed to repeated sub-concussive blows to the head during a game as a college or professional player (Mrazik et al., 2016), over the course of a season as a high school (Tsushima et al., 2016), college (McAllister et al., 2012; Mrazik et al., 2016), or professional (Mrazik et al., 2016) player; (iii) began playing contact football during pre-adolescence as opposed to adolescence and stopped playing after a career in the NFL (Solomon et al., 2016); and (iv) possess differing levels of a cognitive skill or set of cognitive skills that place them at higher risk for severe head injury as either high school (Schmidt et al., 2015) or DI college (Harpham et al., 2014) players. Typically, this research includes one or more RT measures, because of their putative sensitivity to concussive and sub-concussive blows to the head, as part of a comprehensive neuropsychological battery. In addition, neuroimaging techniques have been included in the assessment. To our knowledge, in none of the extant research did the investigators seek to isolate differences between football players and controls in a specific component or set of components in any given speeded mental reaction or the differential effects, if any, of concussive or repetitive sub-concussive blows to the head on these components.

The central goal of our research with football players is to achieve a comprehensive and well-articulated understanding of how their cognitive skills, like their physical skills, may be superior to those of comparably aged non-athletes. A second goal is to determine the extent to which specific cognitive skills, like specific physical skills, differ across positions. Our working assumptions are that (i) football players at the higher levels of competition possess both a set of physical skills that allows them to meet the physical demands placed on them during a game and a corresponding set of cognitive skills that mediates the expression of those physical skills; and (ii) just as there are differences in the physical skills required of players at different positions, there are differences in the cognitive skills required of them to implement those physical skills.

In our first step toward achieving these goals we completed a study using an arrow variant of the Eriksen flanker task (Eriksen and Eriksen, 1974) to determine if DI football players and their male counterparts in the general student population differ in a specific cognitive skill, *interference control* (Wylie et al., 2018). Interference was induced in this task by flanking a left- or a rightward pointing target arrow, shown at visual fixation, on each side with two non-target arrows that pointed either in the same or the opposite direction as the target. Participants were instructed to focus their attention on the target arrow and to respond, as quickly and as accurately as possible, in the direction it pointed. Thus, the flankers provided *response-relevant* information that could either facilitate the response (i.e., pointed in the same or congruent direction) or interfere with it (i.e., pointed in the opposite or incongruent direction). A vast literature has revealed that response speed is slowed and accuracy is reduced when the target and flankers signal alternative responses (reviewed in Wylie et al., 2018). This decline in performance, the Eriksen flanker effect, is thought to reflect the extent to which processing the incongruent flankers interferes with processing the target and executing the response it signals. Individual differences in the size of this effect are considered to reveal variations in how effectively the interference has been inhibited (discussed in Wylie et al., 2018). We found that football players and controls did not differ in their overall RTs or accuracy levels, in their RTs and accuracy levels when the target arrow and flankers were congruent, and in their accuracy levels when the target arrow and flankers were incongruent. However, the size of the flanker effect was smaller in football players as a group than in controls, indicating that they inhibited the interfering effects of incongruent flankers faster than did their non-athlete counterparts. Importantly, players differed by position in their suppression skill. All defensive player groups (linemen, linebackers, backs) and one offensive position group (wide receivers/tight ends) were more efficient at inhibiting flanker interference than were controls. Offensive linemen, running backs, and quarterbacks were not significantly faster than controls. Hence, we found a very specific superiority in *interference control* among football players that was not uniform across players but varied across positions.

As a second critical step toward achieving our goals, for reasons enumerated below, we chose to assess differences in *impulse control*, induced by *response-irrelevant* spatial information, between football players and non-athlete controls using a variant of the Simon task (see review in Simon, 1990). Fast reactive sports, like football, performed in visually dynamic environments place considerable demands on those components of an athlete’s cognitive control system that regulate the rapid selection and inhibition of actions and may not be revealed in overall measures of response speed or accuracy. Among the most challenging demands on these components are those placed on processes that selectively inhibit the production of fast, impulsive reactions that conflict with the execution of more optimal reactions in situations that necessitate this type of inhibitory control. In football, the need to control premature or incorrect response impulses arises from a broad range of on-field events. For example, offensive linemen must suppress impulses to react before a play begins (i.e., commit a false start violation) that can be induced by a defensive player’s feigning quick, explosive movements at the line of scrimmage before the ball is snapped. On defense, linebackers who are preparing to blitz must likewise suppress impulses to react before a play begins (i.e., commit an encroachment violation) that can be induced by the rapid initial steps of offensive players as they shift positions or go into motion before the ball is snapped. Similarly, when the ball is in play, defensive and offensive players alike must control initial impulses to react to an opponent’s use of rapid misdirection movements or motions (e.g., jukes, hitches, counter moves) as they attempt to make a tackle, a block, or maintain coverage on a receiver as well as evade being blocked, tackled, or covered on pass-running routes. Because a first impulse may not be the best response to an evasive movement, a player must minimize its interference with the efficient execution of a desired or correct response by engaging cognitive control mechanisms to inhibit the impulse and prevent its expression.

The capacity to exercise this type of control is commonly considered by football experts (e.g., coaches, scouts) to be among the skills highly talented players possess that cannot be measured directly and can only be inferred from a player’s on-field performance. That is, it is deemed to be an “intangible” or “instinctual” skill by these experts that is not accessible to direct testing. Although seemingly immeasurable to football experts, interest in the cognitive processes that control this type of skill can be traced in Psychology to its beginning as a scientific discipline. Indeed, the foundational research in scientific psychology was devoted to studying processes of this type, with the first doctorate in psychology awarded in 1880 for a study designed to reveal the structure and timing of speeded decision-making using RT methods (i.e., mental chronometry; O’Shea and Bashore, 2012). In the 140 years or so since that pioneering research was undertaken cognitive psychologists (née experimental psychologists) have developed a wide range of highly refined experimental tasks to assess cognitive skills like those required of football players to control motor impulses. This form of control can be investigated and measured directly using response conflict tasks that comprise an important part of cognitive psychology’s developmental history. A particularly powerful task with a rich history is the Simon conflict task, which has the added advantage of measuring the ability to control incorrect response impulses that are triggered by the processing of irrelevant spatial information, thus having the potential to connect to the forms of spatially driven response impulses often produced on the football field.

In the most commonly used variant of the Simon task, individuals are instructed to issue a speeded reaction to a designated feature of a visual stimulus that appears randomly either to the left or to the right of visual fixation along the horizontal meridian. For example, a series of color-filled circles is presented randomly in either the left or the right visual half-field and individuals are instructed to issue a left-hand button press to an orange-colored circle and a right-hand button press to a blue-colored circle, irrespective of the circle’s half-field location relative to visual fixation. Overall mean RTs and accuracy rates in this two-choice task vary across young adults, but are generally issued in the 350–500 millisecond (ms) range with overall accuracy rates around 90–95%. Most importantly, a highly robust and consistent pattern of effects on RTs and accuracy rates is produced by the spatial location of each circle vis-a-vis the signaled response hand, even though spatial location is irrelevant to the decision-making process. This pattern of effects was first reported in a series of papers by J. R. Simon and colleagues in the late 1960s and early 1970s (Simon and Rudell, 1967; Simon, 1969; Craft and Simon, 1970; Simon et al., 1973; Simon et al., 1976; reviewed in Simon, 1990).

When, in our example, the circle appears in the left visual half-field reactions are fastest and most accurate when its color also signals a left-hand response. That is, performance is facilitated when the spatial location and the response-relevant feature of the stimulus are *corresponding*. In contrast, when the response hand activated by the spatial position of the circle conflicts with the response hand signaled by the color of the circle, reactions are slowed significantly and are more error prone. Slowing is on the order of 35–40 ms and error rates increase by about 8–10% among young adults in the general population. Thus, performance is degraded, marked by a slowing in response speed and an increase in errors when the stimulus location and the response signaled by the imperative feature of the stimulus are *non-corresponding*. The magnitude of these performance costs (coined the *Simon effect* by Hedge and Marsh, 1975) provides a quantitative index of an individual’s susceptibility to activating incorrect impulsive motor reactions as well as individual differences in the time needed to inhibit this activation. On a neurophysiological level, the *Simon effect* is associated with an initial short latency activation of motor cortex contralateral to the hand located in the hemi-space ipsilateral to the visual hemi-field in which the imperative stimulus appeared (e.g., right hand/right side of the body, right visual hemi-field) that is followed by subthreshold electromyographic muscle activations in that, incorrect response, hand (Burle et al., 2002, 2014; van Wouwe et al., 2014).

An extensive literature has revealed that resolution of the conflict induced by spatial non-correspondence in the Simon task is accomplished by cognitive control mechanisms, specifically inhibitory control processes, in frontal-subcortical circuitries that are engaged to suppress the activation and execution of incorrect, impulsive motor responses (Ridderinkhof, 2002; Forstmann et al., 2008a,b; Wiecki and Frank, 2013). The most-widely accepted explanation of how this conflict is induced and resolved is found in the dual-process activation suppression (DPAS) model (Ridderinkhof et al., 1995; Mattler, 2005; van den Wildenberg et al., 2010). Facilitation or conflict are thought to arise from the concurrent activation of automatic and controlled processes. Automatic processing of the spatial location of the stimulus is faster than controlled processing of the response-relevant feature of the target and produces an initial activation of the corresponding response hand that either facilitates the reaction signaled by the target (i.e., a response by the *corresponding* hand) or conflicts with the reaction signaled by the target (i.e., a response by the *non-corresponding* hand). When the spatial location of the imperative stimulus and its response-relevant property are non-corresponding, activation of the incorrect response must be suppressed by cognitive control mechanisms to prevent a premature erroneous response, which interferes with activation of the correct response and thereby slows its execution. Suppression increases in strength over time and does not achieve maximum effectiveness until late in processing when the response selection decision is being made. Thus, the size of the *Simon effect* provides an objective index of the proficiency with which an individual’s cognitive control system inhibits impulsive motor system activation induced by response conflict.

A large body of research has emerged over the past two to three decades that has revealed the power of the prototypical Simon task and its variants to identify deficits in *impulse control* associated with different types of neurological [e.g., Parkinson’s disease: van Wouwe et al., 2016; Servant et al., 2018; Tourette’s: Wylie et al., 2013] or psychiatric [Attention Deficit Hyperactivity Disorder (ADHD): Ridderinkhof et al., 2005] disorders. In contrast to this large literature, we are aware of only a small number of studies designed with the express purpose of assessing this type of control in highly skilled athletes, who may possess superior control, using variants of the conventional Simon task [*bowling* (Braem et al., 2015); *boxing* (Ottoboni et al., 2015); also see Davranche et al. (2009) for the influence of physical exercise intensity and response effector (foot) on the properties of *Simon effect* in elite *white-water kayakers*]. This relative scarcity of basic research may be reflective of controversy in cognitive sports science about the degree to which basic cognitive skills assessed using conventional laboratory tasks, like the Eriksen flanker task and the Simon task, rather than sport-specific laboratory tasks, like action anticipation tasks that require speeded reactions to videos of players simulating game-like actions, are predictive of the on-field performance of athletes at any level, particularly those who must utilize their cognitive skills in visually dynamic environments (for review see Ward et al., 2017). The greater intuitive and ecological appeal of the latter may have shifted interest in its direction.

There is a growing literature, nonetheless, in which differences in cognitive skills between athletes and non-athlete controls or between open-sport athletes and closed-sport controls are being assessed using a variety of conventional laboratory tasks that include RT as a primary dependent measure [e.g., *professional athletes* (*mixed*), Bianco et al., 2017a (continuous performance); *baseball*, Kida et al., 2005 (go/nogo); Nakamoto and Mori, 2008 (go/nogo)/Nakamoto and Mori, 2012 (coincident timing); Yamashiro et al., 2013 (go/nogo); Yamashiro et al., 2015 (go/nogo); *badminton*, Wang et al., 2017 (flanker); *boxing*, Bianco et al., 2017b (go/nogo); *fencing*, Bianco et al., 2017b (go/nogo); *martial arts*, Sanchez-Lopez et al., 2014 (continuous performance); *table tennis*, Guo et al., 2017 (go/nogo); Hung et al., 2004 (cued two-choice RT); *tennis*, Overney et al., 2008 (coherent motion); Wang et al., 2013 (stop-signal)]. Impetus for this research effort has certainly been provided by the results of a meta-analysis by Voss et al. (2010) who found that collegiate and professional athletes, particularly those who compete in visually dynamic environments (identified by the authors as interceptive sports), outperformed non-athletes in conventional laboratory tasks that assess processing speed and distraction control. Moreover, the appeal of conventional laboratory tasks with long histories is that they are well-characterized methodologically, yield reliable patterns of factor effects in the basic task and its variants in non-athletes against which the performance of athletes on these tasks can be compared, and provide a rich conceptual framework within which to interpret any differences that may be found between athletes and controls. In addition, they are typically easy to administer. Of course, the critical proof of their value is whether possession of a superior basic cognitive skill, for example *interference control* revealed in an athlete by the Eriksen flanker task, predicts superior performance by that athlete in situations on the field when that type of control must be exercised.

In our view, conventional laboratory research provides the essential antecedent work for establishing the degree to which fundamental cognitive skill differences exist between football players and controls, and lays the necessary groundwork for research designed to determine if linkages do exist between cognitive skills assessed in conventional laboratory tasks, cognitive skills assessed in sport-specific laboratory tasks, physical skills, and on-field performance. The meta-analysis by Voss et al. (2010) and the emerging body of comparative RT research suggests that this line of inquiry is likely to be of value. There are few tasks in the cognitive sciences that are as thoroughly studied and widely used as the Simon task. Hence, our choice. Recall, Voss et al. (2010) found overall faster processing speeds in athletes than in non-athlete controls across a range of tasks. However, the RT literature is mixed. That is, athletes have been found to be faster than (e.g., Kida et al., 2005; Yamashiro et al., 2013; Ottoboni et al., 2015; Bianco et al., 2017a,b; Guo et al., 2017; Wang et al., 2017), comparable to (e.g., Wang et al., 2013; Sanchez-Lopez et al., 2014; Yamashiro et al., 2015; Wylie et al., 2018), or slower than (Ottoboni et al., 2015) controls in their response speeds [see Nakamoto and Mori (2008) for task-specific varied outcomes]. Our finding that DI collegiate football players and controls did not differ in either overall RT or accuracy on the Eriksen flanker task led us to predict that there would be no overall differences between the two groups on either measure in the Simon task. However, given the considerable demands that football places on a player’s skill at controlling initial impulses to react to an opponent’s efforts to induce such impulsive reactions, we expected collegiate football players to be more efficient than non-athlete controls at controlling the impulse to respond to the irrelevant spatial location of the imperative stimulus (i.e., to have a smaller *Simon effect*). That is, we predicted that football players as a group would excel at a specific cognitive skill, *impulse control*, as they did at *interference control*. Finally, the size of our football player sample (*n* = 276) permitted us to do exploratory comparative analyses of differences in motor *impulse control* between offensive and defensive players as well as between players at different positions on the offensive and defensive sides of the ball.

## Materials and Methods

### Participants

Data were collected from 276 male collegiate football players (mean age 19.8 ± 1.5 years) and 32 male controls from the general student population (mean age 19.6 ± 1.7 years). Football players were all on the current team rosters of five National Collegiate Athletic Association (NCAA) DI football programs. Controls were recruited from the general university population at the University of Northern Colorado and interviewed to confirm no history of participation in collegiate sports. They received course credit for their participation in the study. None of the football athletes were in an active concussion protocol at the time of testing or had experienced a blow to the head that kept them from physical activity within the 3 months prior to testing. Controls had no history of head injury. All participants had normal or corrected-to-normal vision, as indicated by self-report. Informed written consent was obtained from individual controls and from athletic programs at each university where testing was conducted on behalf of all of its athletes. In written agreements with the athletic departments, football programs assumed all responsibilities for athlete consent to complete the protocol, and athletes were informed of the protocol, consented orally, and participated voluntarily, but were not required by the athletic department to sign a written consent^2^. Per agreements with the athletic programs, we were allowed to use, analyze, and report on athlete data provided that the identity of the university and athletes remained de-identified. The study and consenting procedures were reviewed and approved by the Institutional Review Boards at the University of Louisville and University of Northern Colorado.

### Simon Task and Procedures

The *Simon task* was administered on a MacMini with a 17-inch Dell monitor placed approximately 1 m in front of the participant. It was one of a series of cognitive tasks completed by the participants. Programming and administration of the tasks were accomplished using PsychToolbox and Matlab software tools (MathWorks, 2014), which interfaced with an RB series response button box to register responses with 2–3 ms RT resolution (Cedrus, Incorporated^3^). The Simon task was initiated when a small, centrally located white square (0.5 cm sides), shown against a dark gray-colored background, appeared on the monitor screen and remained visible on the screen for the entire task. Within 1000 ms following the initial appearance of the fixation square, a blue or an orange circle appeared to the left or to the right of the fixation square. The circle remained on the screen for 250 ms and then disappeared. Participants were given 1000 ms from the onset of the circle to respond. When this time limit was exceeded by 50 ms the next colored circle appeared; that is, the interstimulus interval was 1050 ms. The end of the task was indicated by the offset of the fixation square and the appearance of printed instructions, centered on the computer screen, that the task was completed.

Participants were instructed to respond to the color of the circle according to a pre-assigned color-response hand mapping linking each color to a button press using the index finger on each hand. The two colors were luminance matched and the color-response hand mapping was invariant across subjects (orange circle, left-hand button press; blue circle, right-hand button press). Our decision to keep the mapping constant across subjects was determined by a pilot study in which data were collected from 10 subjects, none of whom participated in the experimental study, to confirm that RTs to the two colors did not differ. The response device was positioned in front of the participant so that the left and right index fingers were placed, respectively, on the far left and far right response buttons of its horizontal 7-button panel. The blue and orange circles were presented pseudo-randomly; that is, with the constraint that they appeared with equiprobability across the task. Participants were encouraged to focus visual attention on the fixation square and to respond as quickly and as accurately as possible when the circle appeared.

The *Simon effect* was elicited by the spatial correspondence between the response impulse activated by the half-field in which the circle appeared and the correct response hand signaled by its color. This factor, *Spatial Correspondence*, had two levels, *Corresponding* (*Cs*) and *Non-corresponding* (*Nc*) trials. On *Cs* trials, the circle appeared in the visual half-field on the same side as the response signaled by the circle’s color (e.g., an orange circle calling for a left-hand response appeared in the left visual half-field). Thus, the response impulse activated automatically by the circle’s spatial location matched the correct response hand signaled by the circle’s color. On *Nc* trials, the circle appeared in the visual half-field on the opposite side of the response hand signaled by the circle’s color (e.g., an orange circle calling for a left-hand response appeared in the right visual half-field). Thus, on *Nc* trials the response impulse activated automatically by the circle’s spatial location conflicted with the correct response hand signaled by the circle’s color. Like the color of the circle, *Cs* and *Nc* trial types occurred pseudo-randomly throughout the task. In total, participants completed 30 practice trials followed directly by 100 experimental trials, equally divided between the different circle color and correspondence type combinations.

### Data Analyses

Mean RTs were calculated for correct response trials across *Cs* and *Nc* trials. We also calculated mean accuracy rates separately for the two trial types. However, because accuracy rates are not normally distributed in choice reaction tasks, we analyzed means of square root-transformed accuracy rates (McDonald, 2014). In addition, analyses were completed on the *Simon effect*, derived by subtracting mean RT and accuracy rates for *Cs* trials from those for *Nc* trials. Smaller Simon effects on RT and accuracy rates indicate higher proficiency at controlling incorrect response impulses. The mean RT and transformed accuracy measures were first analyzed separately using repeated-measures analysis of variance (ANOVA) to determine the main and interactive effects of *Spatial Correspondence* (*Cs, Nc*) and *Group* (*football player, general student control*). ANOVAs of the *Simon effect* assessed differences in its magnitude between football players and controls. We then re-analyzed the dependent measures within the football athletes to assess the effect of *General Position* (*Offense, Defense*) followed by more specific analyses to address potential performance differences in *Specific Positions* within each group of offensive [Quarterback (*QB, n* = 27), Running Back (*RB, n* = 30), Wide Receiver (*WR, n* = 35), Tight End (*TE, n* = 16), Offensive Lineman (*OL, n* = 48)] and defensive [Defensive Lineman (*DL*, n = 37), Linebacker (*LB, n* = 30), Defensive Back (*DB, n* = 45)] player groups (note: a small subgroup of eight punters/kickers was excluded from the last two sets of analyses as their positions are not typically associated with Offense or Defense).

Because our working hypothesis is that football players control spatially driven incorrect impulsive responses more proficiently than do their non-athlete counterparts (i.e., the size of the *Simon effect* will be smaller among football players than controls), our test of this hypothesis was one-sided. We had no such directional hypotheses for our comparison of offensive and defensive football players. Hence, this test was two-sided. To provide additional quantification of the strength of these effects for each analysis, we report effect sizes (Cohen’s *d*) and associated 95% confidence intervals (0.95 CI) as well as Bayes factors (Wagenmakers, 2007; Rouder et al., 2009; Wetzels et al., 2011; Jarosz and Wiley, 2014). As a general rule of thumb, Cohen’s *d* values (positive or negative in direction) less than 0.2 are considered to be small, values from 0.2 to 0.5 to be small to medium, values from 0.5 to 0.8 to be medium to large, values from 0.8 to 1.0 to be large to very large, and values greater than 1.0 to be very large. The Bayes factor (BF_+0_) provides the likelihood or odds favoring the alternative hypothesis (i.e., the experimental and control groups differ) over the null hypothesis (i.e., the groups do not differ). Values greater than 1.0 favor the alternative hypothesis that the experimental and control groups differ. The larger the departure from 1.0, the higher the confidence that the difference is actual. As a general rule of thumb, a Bayes factor of 1–3 provides anecdotal evidence, 3–10 substantial evidence, 10–30 strong evidence, 30–100 very strong evidence, and >100 decisive evidence in favor of the alternative hypothesis. Values in the opposite direction (e.g., 1/3–1, 1/10–1/3 … 1/100–1/30, <100) provide increasing support for the null hypothesis. We used the software JASP (JASP Team, 2018) and default priors (*r* = 0.707; also see Rouder et al., 2009) to compute Bayes factors.

## Results

### Comparison of Collegiate Football Players and Controls

**FIGURE 1:**
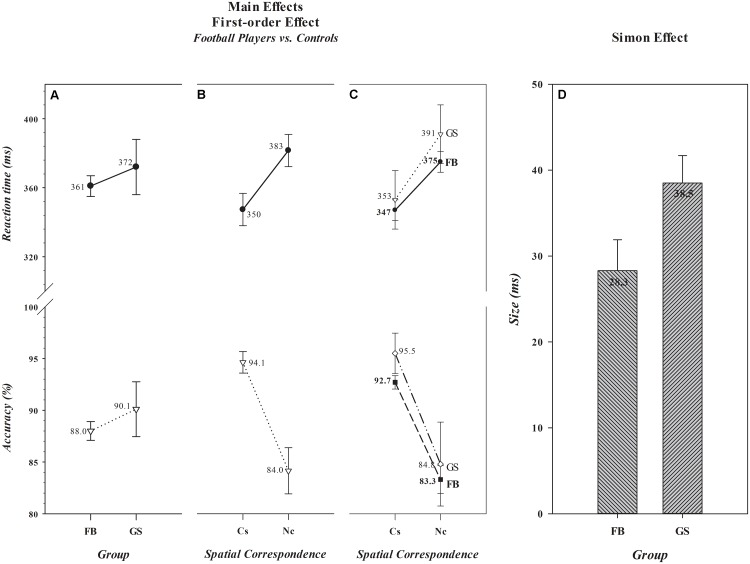
Absence of main effects of *Group* and presence of main effects of *Spatial Correspondence* for reaction time (RT) (in ms) and accuracy (in percent correct) are shown, respectively, in **A**,**B**. The values associated with each data point are shown by each point. The size differences in the *Simon effect* (in ms) that produced the significant *Group × Spatial Correspondence* interaction are illustrated in Panels **C**,**D**. In **D**, the absolute size (in ms) of the effect for each group is shown in the bar for that group. Error bars represent the 0.95 confidence interval. FB, football athletes; GS, general student control; Cs, corresponding trials; and Nc, non-corresponding trials.

As can be seen in panel A of **Figure 1**, overall mean RTs and accuracy rates did not differ between football players (361 ms, 88.0%) and controls (372 ms, 90.1%) [*Group, F*(1,306) (RT, *F*= 1.71, *p*= 0.193) (Acc, *F*= 2.21, *p*= 0.138)]. It can also be seen in panel B that a significant *Simon effect* was produced on both dependent measures [*Spatial Correspondence, F*(1,306) (RT, *F*= 319.29, *p*< 0.001) (Acc, *F*= 306.00, *p*< 0.001)]. Responses were about 33 ms slower and 10% less accurate on *Nc* than on *Cs* trials. However, the size of the *Simon effect*, illustrated in panels C and D, differed between the two groups for RT, but not for accuracy [*Group × Spatial Correspondence, F*(1,306) (RT, *F*= 7.23, *p*= 0.008) (Acc, *F*= 0.31, *p*= 0.576)]. The *Simon effect* on RT was significantly smaller among football players (28.3 ms) than controls (38.5 ms) (*t*(306) = -2.71, *p*= 0.007), a mean difference [-10 ms, 0.95 CI (-18, -4)] that was a medium to large effect size [Cohen’s *d* = -0.51, 0.95 CI (-0.87, -0.14)]. The Bayes factor analysis provided strong evidence (BF_+0_ = 10.5) in favor of the alternative hypothesis. Thus, we see analytic convergence supporting the prediction that spatially driven activation of incorrect response impulses caused significantly less reduction of response execution speed among football players than controls, indicating that *impulse control* is more proficient among players than controls.

### Comparison of Offensive and Defensive Football Player Groups

**FIGURE 2:**
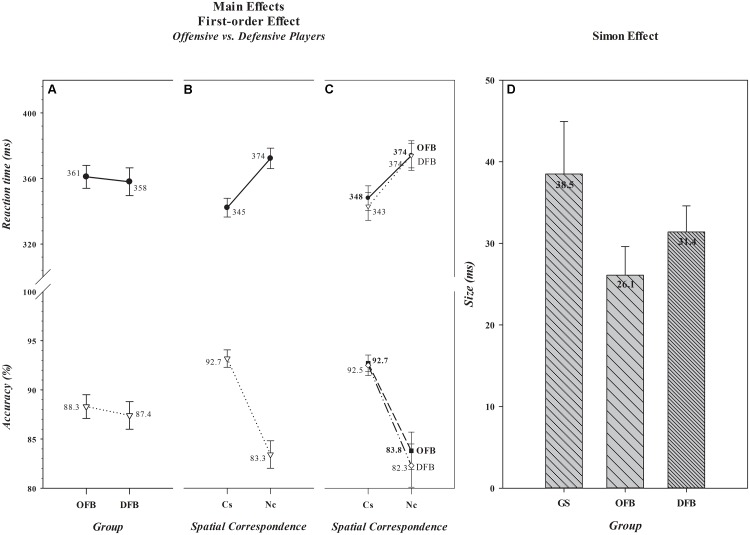
Absence of main effects of *Group* and presence of main effects of *Spatial Correspondence* for RT (in ms) and accuracy (in percent correct) for the comparison between offensive and defensive football players are shown, respectively, in **A**,**B**. The values associated with each data point are shown by each point. The size differences in the *Simon effect* (in ms) that produced the significant *Group × Spatial Correspondence* interaction when offensive and defensive players were compared against general student controls are illustrated in **C,D**. In **D**, the absolute size (in ms) of the effect for each group is shown in the bar for that group. Error bars represent the 0.95 confidence interval. OFB, offensive football players; DFB, offensive football players; GS, general student control; Cs, corresponding trials; Nc, non-corresponding trials.

The overall mean response speeds and accuracies of offensive [*n* = 156 (361 ms, 88.3%)] and defensive [*n* = 112 (358 ms, 87.4%)] players, illustrated in **Figure 2A**, were nearly identical [*Group, F*(1,266) (RT, *F*= 0.30, *p*= 0.587) (Acc, *F*= 0.72, *p*= 0.396)]. As was the case in the comparison between football players and controls, a very robust *Simon effect*, depicted in **Figure 2B**, was evident in this comparison [*Spatial Correspondence, F*(1,266) [RT, *F*= 530.77, *p*< 0.001) (Acc, *F*= 205.62, *p*< 0.001)]. Responses were about 29 ms slower and 9% less accurate on *Nc* than on *Cs* trials. Of greatest interest, however, is that the magnitude of the effect differed between offensive and defensive players on RT, but not on accuracy [*General Position × Spatial Correspondence, F*(1,266) (RT, *F*= 4.46, *p*= 0.036) (Acc, *F*= 0.79, *p*= 0.374)]. Specifically, the magnitude of the effect on RT was significantly smaller among offensive (26.1 ms) than defensive (31.4 ms) players, [*t*(266) = -2.20, *p*= 0.029], a mean difference [-5 ms, 0.95 CI (-10, -1)] that was a small to medium effect size [Cohen’s *d* = -0.26, 0.95 CI (-0.50, -0.02)] with a 0.95 CI that did not encompass, but closely approached, zero. The Bayes factor analysis provided anecdotal evidence that the *Simon effect* differed between offensive and defensive position groups (BF_+0_ = 1.1). Thus, the analytical evidence is suggestive but not decisive, indicating that some reservation be exercised in interpreting the difference between these broad position groups.

A separate ANOVA comparing offensive and defensive player groups to controls revealed, as depicted in **Figures 2C,D**, that the *Simon effect* on RT was smaller in offensive players than in controls and that defensive players showed a numerical, but statistically less reliable, advantage [*Group, F*(2,297) = 5.96, *p*= 0.003; Dunnett’s *post hoc* tests: Offensive vs. Controls, *p*= 0.001, Defensive vs. Controls, *p =*0.059]. For offensive players, the mean difference [-12 ms, 0.95 CI (-21, -4)] was a medium to large effect size [Cohen’s *d* = -0.57, 0.95 CI (-0.96, -0.19)], and for defensive players, the mean difference (-7 ms, 0.95 CI (-14, -1)] was a small to medium effect size [Cohen’s *d* = -0.41, 0.95 CI (-0.81, -0.01)]. However, the CIs for defensive players closely approached zero. The Bayes factor analyses provided substantial evidence that the *Simon effect* was smaller in offensive players than in controls (BF_+0_ = 19.8), but only anecdotal evidence that the effect was smaller in defensive players than in controls (BF_+0_ = 2.6). Thus, there is converging analytic support of the hypothesis that offensive position players as a group have better control over spatially driven incorrect response impulses than do controls. However, the analytic outcomes are suggestive but not decisive that defensive players as a group have an advantage over controls, again indicating reservation in interpreting this difference.

### Comparison of Offensive and Defensive Football Position-Groups

**FIGURE 3:**
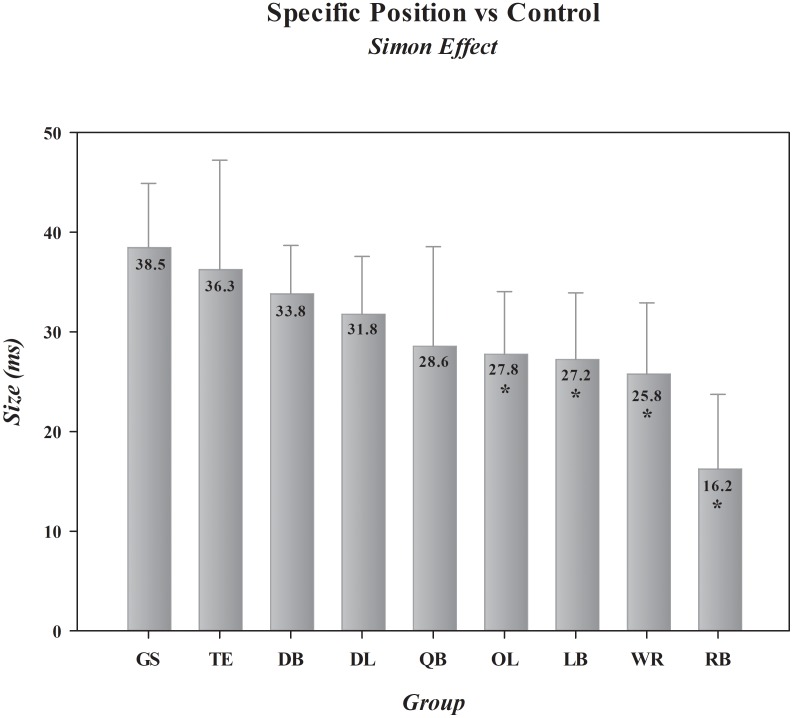
Comparative sizes of the *Simon effect* in general student controls and in football players differentiated by position. ^∗^ indicates statistical convergence indicating that an actual difference exists between a particular position-group and the general student control group. Error bars represent the 0.95 confidence interval. GS, general student control; QB, quarterback; OL, offensive lineman; RB, running back; WR/TE, wide receiver/tight end; DB, defensive back; DL, defensive lineman; LB, linebacker. The absolute size of the effect (in ms) for each group is shown in the bar for that group.

In this final set of analyses, we compared specific offensive (*QB, RB, WR, TE, OL*) and defensive (*DL, LB, DB*) position-groups to determine if the *Simon effect* differs in magnitude between players who are tasked with unique demands on the football field. We then compared each of these position-groups against the control group to identify which position-group or groups display advantages over their non-athlete counterparts in *impulse control*. Separate one-way ANOVAs on the magnitudes of the *Simon effect* on RT and on response accuracy revealed that the size of the effect varied across position-groups for RT, shown in **Figure 3**, but not for accuracy [*Specific Positions, F*(8,291) (RT, *F*= 3.36, *p*= 0.001) (Acc, *F*= 1.29, *p*= 0.249)]. Dunnett’s *post hoc* comparisons of the *Simon effect* on RT between the football position-groups and the control group revealed that two offensive position-groups, *RB* [*p <*0.001; mean difference = -22 ms, 0.95 CI (-32, -13)] and *WR* [*p*= 0.027; mean difference = -13 ms, 0.95 CI (-22, -3)], had significantly smaller effects on RT than did the other position-groups. The group of 30 *RBs* showed an average *Simon effect* of just 16 ms, nearly 60% smaller than the size of the effect measured in controls. Of note, two additional position-groups, one offensive, *OL* [*p*= 0.052; mean difference = -11 ms, 0.95 CI (-20, -2)], and one defensive, *LB* [*p*= 0.070; mean difference = -11 ms, 0.95 CI (-20, -2)], narrowly missed Dunnett’s significance threshold. The remaining position groups, *TE* [*p*= 0.782, mean difference = -2 ms, 0.95 CI (-14, 9)], *DB* [*p*= 0.493, mean difference = -5 ms, 0.95 CI (-12, 3)], *DL* [*p*= 0.314, mean difference = -7 ms, 0.95 CI (-15, 2)], and *QB* [*p*= 0.136; mean difference = -10 ms, 0.95 CI (-21, 1)], despite showing numerically smaller effects, did not differ statistically from controls. These comparisons produced a range of effect sizes across positions, from effect sizes with CIs encompassing zero [*TE*, Cohen’s *d* = -0.12, 0.95 CI (-0.72, 0.48); *DB*, Cohen’s *d* = -0.28, 0.95 CI (-0.73, 0.18); *DL*, Cohen’s *d* = -0.38, 0.95 CI (-0.85, 0.10); *QB*, Cohen’s *d* = -0.46, 0.95 CI (-0.97, 0.07)], to medium to large effect sizes with non-zero encompassing CIs [*OL*, Cohen’s *d* = -0.53, 0.95 CI (-0.98, -0.07); *LB*, Cohen’s *d* = -0.63, 0.95 CI (-1.13, -0.11); *WR*, Cohen’s *d* = -0.65, 0.95 CI (-1.14, -0.15)], to a very large effect size among *RBs* [Cohen’s *d* = -1.17, 0.95 CI (-1.70, -0.62)].

The Bayes factor analysis comparing the odds that the *Simon effect* was smaller among specific position-groups than among controls provided evidence favoring this hypothesis that ranged from inconclusive (*QB*, BF_+0_ = 0.1; *TE*, BF_+0_ = 0.4; *DB*, BF_+0_ = 0.8), to anecdotal (*DL*, BF_+0_ = 1.3), to substantial (*OL*, BF_+0_ = 4.6; *LB*, BF_+0_ = 6.3; *WR*, BF_+0_ = 9.5), to decisive (*RB*, BF_+0_ = 1717.7). Thus, we see analytic convergence supporting position-specific differences in *impulse control*, with *RB*s showing a decisive advantage over controls, *WR, OL*, and *LB* position-groups showing compelling advantages over controls, and *QB, TE, DB*, and *DL* position-groups showing no advantage.

## Discussion

Consistent with our findings for the Eriksen flanker task (Wylie et al., 2018) and for a subset of RT studies with other athletes (e.g., Wang et al., 2013; Sanchez-Lopez et al., 2014; Yamashiro et al., 2015), football players and non-athlete controls showed similar overall mean RTs and accuracy rates in the Simon task. This pattern of global effects suggests that insight into differences in speeded decision-making processes between elite athletes and their non-athlete counterparts is not necessarily provided by global performance measures in basic two-choice RT tasks. However, this pattern of effects does indicate that football players and controls approached the task similarly in how they balanced speed with accuracy when making their response execution decisions. Most critically, a robust *Simon effect* emerged. Responses, collapsed across groups, were slower and less accurate on *Nc* than on *Cs* trials. That is, collectively, conflict induced by the initial response impulse degraded the quality of the goal-driven response. This replication of the classic pattern of performance costs which constitute the *Simon effect* set the stage for a powerful test of the hypothesis that collegiate football players are more proficient than student controls in their ability to execute speeded reactions while controlling spatially activated response impulses. Support was found for this hypothesis. Moreover, as was the case for the Eriksen flanker task, the magnitude of the *Simon effect* differed between player position-groups. However, where defensive players and wide receivers/tight ends were statistically superior to controls in *interference control*, a subset of offensive players (*RB, WR, OL*) and one defensive position-group, linebackers, were statistically superior to controls in *impulse control*. Specifically, the superiority among this group of running backs is dramatic and statistically decisive, and among wide receivers, linebackers, and offensive linemen is compelling.

It is important to note that differences in *impulse control* between football players and controls and between different positions among football players were restricted to speed, as was the case for *interference control*. Football players and controls exercised comparable control of overt impulsive motor errors, each showing about a 10% increase in these errors on conflict (i.e., *Nc*) trials. Thus, executive control systems of both groups were captured to the same extent by strong motor impulses. Correct responses on the remaining 84% of conflict trials were slowed significantly and consistently, an expected effect on execution speed that is thought to be secondary to the initial activation and subsequent inhibition of the incorrect response impulse. However, unlike accuracy, the magnitude of the slowing induced by conflict differed between the two groups. The time taken by non-athlete controls to suppress activation of the incorrect motor impulse and execute the correct response added an average of about 38 ms to their average RT, a cost tightly aligned with an extensive research literature (see reviews in Hommel, 2011; Proctor et al., 2011). In contrast, the cost incurred by football players as a group was about 28 ms, a 26% speed advantage in suppressing an incorrect response impulse and executing a correct response. A simple metric for putting this speed difference in perspective is found in the 40-yard dash. A 26% advantage in the 40 is equivalent to the difference between a 4.2 s and a 5.2 s time. In a race between a player with 4.2 speed and a control with 5.2 speed the player will have crossed the finish line at the same time the control has just passed the 30-yard line. Not a subtle difference in speed. Similarly, an advantage of this magnitude in *impulse control* speed is not subtle. This pattern of results suggests that, collectively, the decided speed advantage a subset of collegiate football players possesses in *impulse control* is mediated by a uniquely proficient component of the brain’s executive control system and is not simply an expression of more proficient global activation of this system.

Resolution of the conflict induced by spatially activated response impulses in the Simon task is accomplished by cognitive control mechanisms in frontal–basal ganglia circuitries that are engaged to inhibit unwanted or errant motor impulses (e.g., Mink, 1996; Forstmann et al., 2008a,b; Jahfari et al., 2011; Ridderinkhof et al., 2011; Wiecki and Frank, 2013; van Wouwe et al., 2017). Indeed, neurological disorders that disrupt these circuitries, such as Parkinson’s disease (e.g., van Wouwe et al., 2016), Tourette’s syndrome (e.g., Wylie et al., 2013), and ADHD (Ridderinkhof et al., 2005), exacerbate conflict costs in the Simon task. Conversely, healthy young adults who are more proficient then their counterparts at suppressing response impulses show enhanced activation in prefrontal areas that are associated with these circuitries and tightly connected to inhibitory control processes (e.g., Forstmann et al., 2008a,b; Jahfari et al., 2011). Thus, the finding that college-level football players are more proficient at controlling response impulses than comparably aged controls is suggestive of the existence of fundamental differences in the structural or functional neural circuitry underlying *impulse control*. A fascinating next step is to establish empirical linkages between *impulse control* capabilities and functional brain activity in these players (see our discussion below).

It is particularly interesting that positional differences in *impulse control* capabilities emerged, as they did for *interference control*. Recall, however, that the pattern of superiority was largely in the opposite direction. Defensive players were generally superior to offensive players in *interference control*, whereas offensive players were generally superior to defensive players in *impulse control*. While offensive players showed more proficient control over response impulses than did defensive players, one offensive position-group, running backs, showed extraordinary proficiency at controlling these impulses. In fact, running backs slowed an average of just 16.2 ms on trials in which incorrect initial response impulses (i.e., *Nc* trials) were activated, a remarkable 58% reduction in response time slowing compared to non-athlete controls who, recall, slowed an average of 38.5 ms. Returning to our 40-yard dash comparison. A running back with 4.2 speed competing against a control who is 58% slower (i.e., with 6.7 speed) will cross the 40-yard finish line when the control is around the 17-yard line. An astonishing difference in speed. Again, the specificity of this effect among running backs was highlighted by the absence of global RT and accuracy rate differences and tradeoffs between running backs and non-athlete controls. Why might running backs possess such exceptional *impulse control* capabilities?

In the Section “Introduction,” we provided two examples of the importance of offensive linemen and linebackers being proficient at controlling impulsive motor reactions to quick movements on the other side of the ball to avoid “offsides” penalties (i.e., reacting across the line of scrimmage before the ball is snapped). While reactions of this sort may be examples of impulsive motor acts, premature reactions do not always reflect failure to inhibit motor impulses, spatially driven or otherwise. The fact that the *Simon effect* is produced by what are generally considered inherent linkages between the spatial location of imperative stimulus information and directional reactions to that information may provide clues about why running backs are particularly skilled at controlling spatially driven response impulses. A running back, advancing the ball against defensive players whose goal is to physically thwart his advance, must execute rapid reactions and counter-reactions to avoid being tackled by a defender. Defensive players can appear in a running back’s visual half-fields unpredictably and instantaneously, triggering a series of impulsive motor reactions that may lead either to spontaneous and effective counter-reactions or to the need to quickly suppress a less optimal response impulse. That is, this significant advantage among running backs may represent a necessary cognitive skill to adapt and execute speeded decisions and reactions within a rapidly changing and spatially dynamic environment. Success as a running back unquestionably depends, certainly to an important extent, on the ability to avoid would-be tacklers. Response conflicts that induce delays in executing split-second response decisions, even on the order of tens of milliseconds, can have significant impact on the football field. The ability to execute exceptional control over motor impulses and select optimal reactions with minimal conflict from these impulses may be a requisite cognitive “intangible” for a running back’s success at executing the most effective and decisive reactions. It is also worth noting that wide receivers, whose reduction in the size of the *Simon effect* approached being very strong, quite often run with the football after making a catch. Thus, they effectively become running backs. Hence, *impulse control* skills may contribute importantly to their proficiency as runners after they have caught the ball. Players at both positions must also be able to block effectively, which entails maintaining good position on pass blocking (running backs) and when blocking in the open field (running backs, wide receivers) by minimizing impulsive responses to evasive moves by defenders.

Offensive linemen and linebackers also demonstrated strong skill at inhibiting motor impulses vis-à-vis their non-athlete counterparts. As the quarterback is calling the signals before the ball is snapped offensive linemen must maintain their stance at the line of scrimmage and refrain from even slight flinches, quite often when brief, quick explosive movements are being made by defensive players feigning a rush or a blitz. After the ball is snapped they must maintain stable blocking positions (i) on pass plays that allow them to move their feet very quickly into the best position to block rushing defenders who are making a variety of deceptive moves to avoid being blocked; and (ii) on plays that require them to block off the line of scrimmage in more open space where defenders have greater latitude in the types of evasive moves they can make. On the defensive side of the ball, linebackers are often called upon to blitz the quarterback. To do so most effectively, they must time the start of their blitz to the snap of the ball, not reacting to offensive players going in motion or any other factors extraneous to the actual snap of the ball. It may be the case that linebackers with high levels of *impulse control* are better able to achieve this timing. In addition, they must be proficient tacklers in open space when they are exposed to the deceptive moves of ball carriers, which requires them to inhibit impulsive reactions to these moves. Moreover, they must be resistant to misdirection plays by the offense that are designed to make them react in the opposite direction the play is going, both slowing them down in their pursuit of the play and making them more vulnerable to being blocked.

Our finding of strong to extraordinarily proficient *impulse control* among a subset of football players is novel and, as such, demands replication. Although replication can establish the reliability of our findings, it cannot establish the existence of a relationship between proficiency in this basic cognitive skill and on-field performance. We have offered speculations about the importance of *impulse control* as a running back runs in the open field or as an offensive lineman pass blocks, but they are just that, speculations. The extent to which possession of this basic cognitive skill impacts a player’s performance on the field must be established empirically, not speculatively. There is probably little disagreement among cognitive (neuro)scientists in this problem area that among the most daunting challenges they confront is determining the extent to which performance on conventional laboratory tasks, like the Eriksen flanker task or the Simon task, translates, if at all, as performance on the field. This is, of course, the gold standard by which coaches, scouts, and player development personnel in football or any other sport will judge the value of this research. From our perspective, a logical empirical and conceptual translational nexus exists between foundational research designed to identify differences between football players (more broadly conceived, athletes) and non-athlete controls in basic cognitive skills assessed via conventional, well-developed laboratory tasks, the articulated expression of these basic cognitive skills assessed through sport-specific laboratory-based pictorial, photographic, and video tasks, and the relative influences of basic cognitive and physical skills on a player’s on-field performance. This is, of course, an extraordinarily complex empirical and conceptual trajectory to follow. Our position is that this trajectory emerges from findings generated in basic laboratory studies. Next, we offer our thoughts on follow-up studies of impulse control that may contribute to determining the extent to which this basic cognitive skill contributes to a football player’s on-field performance.

### Impulse Control: Directions for Future Research

Our findings suggest the value of exploring differences in *impulse control* not only between football players and controls, but also between different positions on the football team. An important first step in this process is to extend our analyses beyond mean values. Previous research in our laboratory and others assessing *impulse control* deficits in neurological patients often included distributional analyses to determine differences in the time course of incorrect response impulse activation and subsequent inhibition of that impulse between patients and controls (e.g., Wylie et al., 2010a,b, 2012; van Wouwe et al., 2014, 2016). These analyses uncovered deficits in impulse inhibition among neurological patients that were not evident in analyses of mean values. An example of the promise of this type of analytic approach for identifying differences in *interference control* between expert performers and controls with more precision than is afforded by exclusive reliance on mean values is found in a study of expert pilots by Roberts et al. (2010; summarized in Wylie et al., 2018). Briefly, in trial-by-trial sequential analyses they found that pilots were better able than controls to regulate their responses after conflict trials. Distributional and sequential analyses on data collected from football players in the Simon task are likely to yield differences in patterns of impulse activation and inhibition between football players, particularly running backs, and controls that isolate where in the time course of activation–inhibition and in the trial-by-trial modulation of their responses football players gain their advantage. In so doing, reasonably precise inferences can be drawn about the relative proficiency of running backs, for example, at preventing an initial incorrect impulse, rapidly inhibiting an impulse after it has been initiated, and making subtle adjustments in their control of impulsive reactions across time.

Extending this research to include these analyses is straightforward. There are also numerous critical issues to address that are far less straightforward. One is determining the relationship between players evaluated and/or measured skill levels and their *impulse control*. We did not partition our sample of players, for example, on their judged talent levels by scouts, player personnel, coaches, and recruiters and determine the relationship between these judgements and *impulse control*. Of course, this type of partitioning may be problematic, as first demonstrated by Miles (1931) who asked coaches to evaluate their players by speed and efficiency and found a very modest relationship between their ratings and the players charging times (for a related contemporary discussion of this issue, see Macnamara et al., 2016). We also did not distinguish between players who register significant playing time and those who are primarily backups. One of the challenges in partitioning university-level players in this way is that it fails to account for underclassmen who will eventually become starters. The traditional role of starter versus backup is also unreliable in many college football programs that feature situational rotation of multiple players on the field to execute certain formations or schemes. An important future study would incorporate longitudinal tracking of players to determine how much *impulse control* capability distinguishes players who see significant playing time (i.e., stay on the field during situational substitutions) at the collegiate level, emerge as top level professional prospects, and are drafted into professional football or signed as free agents. Another issue is determining thresholds for *impulse control* that afford advantages or disadvantages on the football field. In this large sample of players, the upper range of *Simon effect* costs on RT extended to 80 ms, with 25% of them showing costs greater than 45 ms, indicating that a sizeable proportion of football players experience considerable slowing of reaction speed when conflicted with incorrect response impulses. Even among positions where the *Simon effect* did not differ from controls, individual differences in the magnitude of the *Simon* effect may still be a critical determinant of performance at that position and predictive of certain on-field performance metrics (e.g., mental mistakes, penalties, statistics). Determining how these cognitive vulnerabilities translate into specific impulsive acts on the field that compromise performance more broadly and at each position is a critically important issue for future investigation. The expression of impulsive motor errors and the slowing response execution speed associated with it are also likely to differ by position (e.g., jumping offsides impulsively, impulsive throws, reacting impulsively to misdirection), and these differences require investigation.

We must caution, however, just as superb speed in the 40 is not sufficient to ensure success on the football field, superb *impulse control* is unlikely to be sufficient to ensure success as a running back, wide receiver, or any other position. Measures of a player’s time in the 40, his vertical jump, his long jump, or the number of times he can bench press 225 pounds constitute indices of his physical hardware, no one of which may be predictive of his on-field performance. Similarly, measures of a player’s speeds at *interference control* and *impulse control* constitute indices of his cognitive hardware, no one of which may be predictive of his on-field performance. Of critical importance is the amount of variance accounted for by each measure, physical or cognitive, in predicting a player’s success at a given position and its relative predictive power vis-à-vis other measures. The present study and our earlier study (Wylie et al., 2018) provide important early pieces of evidence to motivate future studies that address these outstanding questions. There are, of course, fundamentally important issues to be addressed that extend beyond behavioral studies of *impulse control*. These relate to differences between football players, looking carefully at position-specific differences, and controls in the underlying neurocircuitry mediating *impulse control*.

### Neurocircuitry

There may be no better examples of extraordinarily finely tuned cognitive-motor control than what spectators see routinely as they watch elite athletes who perform in visually dynamic sports do what they do, seemingly so effortlessly, on virtually every play. These athletes play with wondrous grace and fluidity not only because they have physical gifts, but also because they have neurological gifts. That is, their brains are exceptionally proficient integrative command centers, processing complex stimulus inputs and generating optimal response outputs with remarkably efficient rapidity and consistency. What is different about how the brains of athletes integrate information and generate motor output commands that makes their remarkable athletic feats so common place? Although a variety of experimental methodologies have been used to provide a “window into the brain” of an athlete, ranging from scalp recordings of brain electrical activity [e.g., event-related brain potentials (ERPs), event-related desynchronization (ERD), event-related synchronization (ERS), brain electromagnetic activity (MEG)] to more direct measures of cerebral activation (e.g., TMS, PET, SPECT, fMRI, DTI)^4^, research findings across these domains have yet to converge on a consensus view of how the brains of highly skilled athletes process information differently than do the brains of non-athletes [e.g., see discussion in Bertollo et al. (2016); see reviews in Debarnot et al. (2014) and Nakata et al. (2010); and meta-analysis in Yang (2015)].

Performing an athletic skill at an elite level requires an essential core of automaticity, whereas modifying that skill during its execution likely requires exquisitely timed transitioning between automatic (bottom-up) responses and controlled (top-down) refinements of those responses in circumstances that demand flexible switching. Indeed, it has been argued that controlled intention may not be necessary for switching like this to occur proficiently; rather, automatic processes may be controlled by top-down processes that do not have to be engaged explicitly (Thompson, 2013). This type of switching is found, for example, on a pass play when the rushing DL slants to the left of the left offensive guard (OG) assigned to block him, the OG recognizes the slant and anticipates a defensive end (DE) looping to his right, reacts to the slanting DL by inhibiting his automatic response (i.e., impulse) to block the slanting DL while at the same time alerting the left offensive tackle (OT) to the slanting DL with a verbal signal (e.g., “go”) and pushing the DL toward the left OT and then quickly re-positioning himself to block the DE before he can get to the quarterback^5^. These “on the fly” adjustments are triggered by highly overlearned stimulus–response relationships that have been established through a combination of many hours of deliberate practice (Ericsson, 2006; Toner and Moran, 2014, 2015), traditional practice, and game experience that provides a context-sensitive “dynamically-updated awareness … where salient features of the environment are tracked and accommodated in an ongoing manner” (Sutton et al., 2011, p. 80; see also Fajen et al., 2008).

Perhaps the first contemporary conceptual framework of the neurocircuitry mediating elite athletic skill to achieve significant currency is the *neural efficiency hypothesis*, which posits that neurocircuits in the expert brain, like that of a high-level athlete, require less activation and, in some circumstances, the engagement of fewer nodes in the circuit to produce high level outcomes [e.g., Del Percio et al., 2008; Guo et al., 2017; see review in Nakata et al. (2010); for a brief insightful commentary on this and related hypotheses, see Callan and Naito, 2014]. However, recent findings have revealed complex patterns of cerebral activation, both increases and decreases, in the brains of non-athletes (e.g., see review in Neubauer and Fink, 2009) and athletes (e.g., Del Percio et al., 2008; Nakata et al., 2010; Vecchio et al., 2012; Debarnot et al., 2014; Yang, 2015) as they perform cognitive tasks that exceed the explanatory reach of this hypothesis. Emanating from this body of research are interesting approaches to modeling neuro-mediation of the type of cognitive processing proficiency and concomitant performance fluidity reflected in our example of the OG that integrate dual-process and attentional control concepts from cognitive science with neural efficiency concepts from cognitive neuroscience [e.g., the *multi-action plan (MAP) model* of Bortoli et al. (2012); the *default-interventionist framework* of Evans and Stanovich (2013a,b), advocated by Furley et al. (2015), as a conceptual framework for sport cognitive science; see also Furley and Wood, 2016]. According to these models, elite athletes optimize their on-field performance by being highly competent at switching very rapidly between automatic and controlled processing to “override prepotent responses,” the latter acting as the overseer of the former (Furley et al., p. 118). Leading proponents of this viewpoint, such as Bertollo et al. (2016), depart from the traditional view that highly skilled athletic performance is optimized when performance achieves complete or near-complete automaticity. The traditional view, they argue, cannot account for the seemingly rapid switching between automatic and controlled processing athletes achieve as they sustain optimal performance by making instantaneous, often very complex, adjustments in their on-going motor reactions to dynamically changing situational conditions (see also Fajen et al., 2008; Toner and Moran, 2014, 2015; Furley et al., 2015). Ericsson (2006) has argued, for example, that proficiency at counteracting automaticity may be critical in developing expertise, in continuing to refine that expertise even when it is very highly developed, and in pushing the boundaries of one’s performance (see also Toner and Moran, 2014, 2015). To our knowledge, no research has been done to assess the cognitive mechanisms mediating how skilled athletes switch between automatic and controlled processing during training and competition (see also Furley et al., 2015).

A prototypical expression of this shared control is found in the Simon task; automatic activation in the direction of the stimulus location and controlled inhibition of that activation when it is incorrect. Hence, this task provides a valuable starting point for assessing differences between athletes and non-athlete controls in the neuro-regulation of automatic activation and controlled inactivation of an incorrect, impulsive response. The neurocircuitry that mediates this type of cognitive control is reasonably well-characterized (e.g., Ridderinkhof et al., 2011; Li et al., 2015) and, as a result, reasonably specific predictions can be made about differences in activation–inactivation patterns between athletes and controls. The study by Forstmann et al. (2008a,b) provides a conceptual–methodological framework for generating and testing such predictions. Guided by the DPAS conceptualization of the processes engaged by the Simon task and utilization of distributional analyses to articulate the time course of the activation and suppression of incorrect impulsive responses (see Introduction for a summary of the DPAS), Forstmann et al. (2008a,b) partitioned their subject sample into poor and good selective response inhibitors. Their fMRI findings revealed that the right inferior frontal cortex (rIFC) was more highly activated in good than in poor inhibitors, their DTI findings revealed that the anterior portion of the fronto-occipital fasciculus (FOF), which conveys visuo-spatial information from the dorsal parieto-occipital and medial parietal cortices to lateral prefrontal areas, was denser in good than in poor inhibitors, and their correlational analyses revealed a strong positive correlation between the two measures suggestive of a tightly linked structure–function relationship between the FOF and the rIFC. Given the putative role of the FOF in the control of elements of higher-order motor behavior and spatial attention, Forstmann et al. (2008a,b) reasoned that proficiency at inhibiting an unwanted impulsive spatial response may be an expression of increased coherency in this pathway. This conceptual–methodological framework supports a straightforward set of predictions about differences in the neuro-regulation of *impulse control* between football players and non-athlete controls. Namely, activation in the rIFC, density of the FOF, and the size of the correlation between these two should be higher in football players, collectively, than in controls, but the existence and magnitude of these differences will vary by position. Based on our (mean) behavioral data, running backs would be expected to have the highest levels of activation in the rIFC, coherence in the FOF, and correlation between the two.

### Closing Comments

Our research has now revealed that DI collegiate football players are more proficient than their non-athlete counterparts in two basic cognitive control skills, *interference control* and *impulse control*. Finding superiorities among a subsample of elite athletes who compete in a visually dynamic sport in basic cognitive skills using conventional laboratory tasks is consistent with and augments the findings of the meta-analysis by Voss et al. (2010), as do the findings from other RT studies we referenced in the Section “Introduction.” Together, this emerging body of work strengthens the argument advanced by Voss et al. (2010) of the value of pursuing this line of research. Indeed, this set of findings provides the empirical bedrock for our position that the fundamental starting point for research aimed at differentiating neurocognitive skills in athletes from non-athletes is assessment of differences between these groups in basic cognitive skills.

An emerging frontier of sports science focuses on understanding the cognitive and neural dynamics of elite athletic performance. Demonstrations that athletes possess unique cognitive capabilities compared to the general population are surprisingly scarce, especially using high precision tools from the cognitive sciences. Quantifying the cognitive “intangibles” of elite athletes represents a critical first step toward optimizing player selection and development. Here we show that a high proportion of DI college football players possess exceptional control over impulsive motor reactions compared to their age counterparts. Moreover, offensive player positions, especially running back, display the most proficient control of response impulses. In fact, their costs to RT fall in a range considerably smaller than is typically reported in studies of this task in young adults (e.g., an impressive 58% reduction among running backs). Thus, the current study provides direct and very suggestive evidence that the speed of controlling impulsive motor reactions may represent an enhanced cognitive “intangible” among elite football players.

## Author Contributions

SW, TB, NvW, and BA contributed to the study conception, design, and data acquisition. All authors (SW, TB, NvW, WvdW, JN, and BA) contributed to the data analysis and interpretation and preparation of the manuscript, and approved the final version to be published.

## Conflict of Interest Statement

The authors declare that the research was conducted in the absence of any commercial or financial relationships that could be construed as a potential conflict of interest.

## Footnotes

1 https://www.ncaa.com/sports/football

2 The reader may be interested in the training regimens of these football players and the impact of differences in them on performance. Recall, that of the over 1,000,000 high school football players, only 2.7% will play at the DI level. These players are recruited by approximately 110 collegiate DI football programs. The recruiting process involves selecting the best football athletes at the high school level. Thus, players at the collegiate level represent a very highly select group of the best football players in the country between the ages of 18–23 years. The governing body of college athletics in the United States, the NCAA, restricts the number of hours each week that college athletes can engage in training. Most programs have similar training regimens for developing players, all of which are intense and require a deep commitment by each player. There is variability in these regimens across the various football programs, however, that is not easily quantifiable. This variation makes it extraordinarily challenging to quantify with any degree of confidence differences in the time and quality of training that translate into differences in on-field performance. Thus, at this point in time skill level is best understood categorically, based on the level of play rather than the type and /or number of training hours. DI football represents the highest level a college player can attain. As our work evolves we hope to tease apart the complex relations between physical skills, cognitive skills, other psychological factors (motivation, persistence, perceived self-efficacy), training techniques, and on-field performance. We are in the incipient stage of this process.

3 https://cedrus.com/rb_series/

4 MEG, magnetoencephalography; TMS, transcranial magnetic stimulation; PET, positron emission tomography; SPECT, single-photon emission computed tomography; fMRI, functional magnetic resonance imaging; DTI, diffusion tensor imaging.

5 We thank Kendall Ryan, formerly a left OG at the University of Northern Colorado, for suggesting this example.
